# Clinical Versus Bacteriological Diagnosis and Quarantine of Diphtheria1Read before the Rochester Pathological Society, January 2, 1902.

**Published:** 1902-04

**Authors:** William L. Conklin

**Affiliations:** Rochester, N. Y.


### Clinical versus Bacteriological Diagnosis and Quarantine of Diphtheria.[1]

By WILLIAM L. CONKLIN, M. D., Rochester, N. Y.

SINCE the advent of antitoxin and the O'Dwyer tube, the feeling of dread with which, in former years, we began the treatment of a case of diphtheria, together with the feeling of despair which too often developed as the case progressed, has largely given way to a well founded hopefulness as to the final outcome.

1. Read before the Rochester Pathological Society, January 2, 1902.

Biggs and Guerard, after a careful review of statistics and published opinions covering the entire period since the introduction of antitoxin in 1892, reach very definite and positive conclusions. They say:

It matters not from what point of view the subject is regarded, if the evidence now at hand is properly weighed, but one conclusion is or can be reached—whether we consider the percentage of mortality from diphtheria and croup in cities as a whole, or in hospitals, or in private practice, or whether we take the absolute mortality for all the cities of Germany, whose population is over 15,000, and all the cities of France, whose population is over 20,000; or the absolute mortality for New York City, or for the great hospitals in France, Germany, and Austria, or whether we consider only the most fatal cases of diphtheria, the laryngeal and operative cases; or whether we study the question with relation to the day of the disease on which treatment is commenced, or the age of the patient treated; it matters not how the subject is regarded or how it is turned for the purpose of comparison with previous results. The conclusion reached is always the same—namely, there has been an average reduction of mortality from the use of antitoxin in the treatment of diphtheria of not less than 50 per cent., and under the most favorable conditions a reduction to one quarter, or even less, of the previous death-rate. This has occurred not in one city at one particular time, but in many cities in different countries, at different seasons of the year, and always in conjunction with the introduction of antitoxin serum and proportionate to the extent of its use.

It would be hard, indeed, to find, in all the history of medicine, another record of results so brilliant as have followed the introduction of serum therapy in the treatment of diphtheria.

It is a fact also, of which as physicians we may justly feel proud, that preventive medicine is receiving more attention today than ever before; and while the problem of the treatment of this dread malady has been studied with such gratifying results, the other and equally important problem of prophylaxis has received the careful attention of sanitarians, both in this country and abroad.

That substantial advance has been made during the last decade is made evident by a careful study of figures recently compiled by W. A. King, chief of vital statistics of the Census Bureau. In 1890 the death-rate in 271 cities of 5,000 and upwards was 21 per 1,000. In 1900, the rate in these cities was 18.8 per 1,000—a decrease of 2.2 per 1,000. In 341 cities of 8,000 and upwards the decrease was 2.4 per 1,000.

This decrease was due very largely to a reduction in the mortality resulting from the diseases of childhood. While there has been an increase of nearly 7,000,000 in the gross population of these cities, there has been as well a decided increase in the average age at death.

During the period to which I have referred many changes have been made in sanitary laws and regulations. But few, if any, of these changes have been of so great importance to both patient and physician as the application of bacteriology to the diagnosis and quarantine of diphtheria. Indeed, I think it may be safely said that as the use of antitoxin is the most important result of the study and investigation of diphtheria in the realm of treatment, so the application of bacteriology to diagnosis and treatment is the most important outcome of its study and investigation in the realm of prophylaxis.

An inovation so radical as the substitution of a bacteriological diagnosis and a bacteriological quarantine for diagnosis and quarantine, which have heretofore been based upon clinical data only, has naturally resulted in much discussion and not a little adverse criticism.

Some of you will remember that at the last meeting of this society, during an interesting discussion of this subject, the query was raised as to whether in view of the imperfections of the more recent method any substantial gain had resulted from its adoption. It is with a conviction and a hope that I have ventured to call your attention to this subject tonight.

My conviction is that the new method, though necessarily imperfect as yet, is far superior to the old, and hence should receive the hearty support of physicians. My hope is that a free discussion from various standpoints may follow the reading of the paper, and that as a result of this discussion much light may be thrown on a subject which is of vital importance alike to the physician and patient.

In no disease is it more important that an accurate differential diagnosis be made as early in the course of the affection as possible. The advantage thus gained is twofold: (1) prompt diagnosis means prompt isolation of the patient and the minimum of risk to others; (2) prompt diagnosis means in the majority of cases the early adoption of such a course of treatment as is most likely to result in speedy and complete recovery. The first advantage growing out of early diagnosis, viz., prompt isolation, can hardly be overestimated.

It is true that the contagiousness of diphtheria is not so well marked as is that of scarlet fever or measles, providing the discharges from nose and throat are properly cared for; still, with the favorable conditions for the development of the disease growing out of crowded and often imperfectly ventilated school rooms, a single unrecognised case may be the starting point of an epidemic. The importance of prompt quarantine is evident when we consider the increase in the number of cases of diphtheria which follows close upon the opening of the schools. This increase in our own city is strikingly illustrated by statistics compiled by the health officer.

For a period of five years, from 1896 to 1900, the average number of cases for July was 13, August 14, September 19, October 42, November 63, December 62. The average was also high for January, February and March, and lower for April, May and June. It is true that other conditions are in some degree responsible for the prevalence of the disease during the winter months, but it would seem evident that infection from mild and consequently unrecognised cases is of all causes by far the most important. Park says: "The most virulent bacillus I have ever found was from a mild case of diphtheria simulating tonsillitis." This being the case, the importance of early recognition and isolation of all cases of diphtheria, whether mild or severe, cannot be too strongly urged.

The second advantage of prompt diagnosis referred to above, viz., the early adoption in the majority of cases of such a course of treatment as is most likely to result in speedy recovery, is indeed great. Whatever method of treatment is adopted, it is evident that if of any value the earlier it is begun the more likely is it to be of avail. Especially is this the case with the serum treatment, for all observers agree that other things being equal its efficacy is in direct proportion to the promptness with which it is used. Unfortunately, when so much is to be gained by the early diagnosis of diphtheria, the clinical picture is often lacking in clearness of outline. Typical cases of pharyngeal diphtheria, with a well defined and rapidly spreading membrane appearing very early in the course of the disease, are the exception rather than the rule. Who has not been at a loss many times to determine from the appearance of an inflamed throat, what the character of the illness was to be? Neither is the degree of constitutional disturbance a safe guide, for not infrequently the onset of an attack of follicular tonsillitis is marked by a greater rise of temperature and a more rapid pulse than are generally found at the beginning of an attack of diphtheria. The clinical differential diagnosis of laryngeal diphtheria and spasmodic croup is often still more difficult.

Reference was made at the last meeting of this society to a local epidemic of diphtheria, which occurred in this city a number of years ago. It was thought that this group of cases originated with one supposed at its inception to be a case of spasmodic croup. It is just such cases of a very serious disease often simulating so closely a malady rarely if ever serious, which are most likely to escape recognition, and escaping recognition most likely to spread the disease.

If, then, the early clinical diagnosis of diphtheria is often difficult, while it is always important, both for the sake of the patient and for the safety of others, is it not wise, imperative even, that we avail ourselves of any aid which the bacteriologist may be able to afford?

Since the investigation of Klebs, in 1883, and Löffler a year later, there has been abundant and constantly increasing evidence that the bacillus, which bears their names, is present in every case of true diphtheria.

Park says:

All the conditions have been fulfilled for diphtheria which are necessary to the most rigid proof of the dependence of an infective disease upon a given microörganism, viz., the constant presence of this organism in the lesions of the disease, the isolation of the organism in pure culture, the reproduction of the essential lesions of the disease in animals and in man by inoculation with pure cultures, the failure to produce all the characteristic lesions of this disease by any other bacteria, and the additional proof of the immunising value of the specific substances developed in animals subjected to injections of diphtheria toxin.

Having, then, a microscopical organism which is always present in diphtheria, on the one hand, and a macroscopical appearance about which there is often nothing pathognomonic, while there is often much which is misleading, on the other hand, shall we not be governed in making a diagnosis by the presence or absence of the microscopical organism rather than by a clinical picture which is so often indistinct and difficult of recognition, especially early in the history of the cases, when accuracy counts for so much?

The objection has been made to the bacteriological diagnosis of diphtheria that it is the physician's prerogative to make his own diagnosis. This is true, but in dealing with cases which may be contagious in character, and hence a menace to the public health, is it not in a special sense his duty to avail himself of every possible aid to correct diagnosis? Further than this—does he not in reality make a tentative diagnosis of diphtheria in every case in which a primary culture is made? Does he not, in fact, call the bacteriologist to his aid much as he might seek the aid of the microscopist in a case of suspected nephritis, or in another of possible malignant growth.

It is true that occasionally the clinical tentative diagnosis of diphtheria may be changed in a day or two to a negative diagnosis, while the bacteriologist on the other hand reports Klebs-Löffler bacilli in the culture, but in view of all we know about the extremes, both of susceptibility and degree of infection in our patients, is it not quite probable that our tentative diagnosis, confirmed by bacteriological examination, is correct?

In comparing the relative merits of a quarantine of diphtheria, based upon bacteriological tests, and a quarantine of diphtheria, based upon clinical conditions, there are certain principles and objections which are worthy of careful consideration.

1. The dependence of diphtheria upon the presence of a specific microörganism, plus an element of susceptibility in the individual which is favorable to its growth and development.

2. The possibility of virulent bacilli remaining in the throat long after all clinical manifestations have disappeared.

3. The object of quarantine, viz.: the prevention and control of epidemics; in other words, the securing so far as possible, the greatest good to the greatest number.

Do not these three facts or principles taken in their logical relationship constitute an argument in favor of quarantine based upon bacteriological rather than clinical data?

The objection is made that a bacteriological quarantine sometimes extends over a period of several weeks, thus causing great inconvenience and expense. This is true, but inconvenience and expense, though matters for regret are not matters for so great regret as follows the needless loss of life.

So careful and competent an observer as Dr. V. A. Moore, of Ithaca, states that a child clinically recovered from diphtheria, though her throat still contained Klebs-Löffler bacilli, was allowed to mingle with her playmates, with a resulting local epidemic and four fatal cases. While, however, bacteriological quarantine means the occasional isolation of a patient for a much longer period than the average time limit of the older method, it means in the long run, a saving of time, for the average of bacteriological quarantine is shorter than that of clinical quarantine.

Chapin states in a recent work on Municipal Sanitation that in 1898, 121 cities and towns in Minnesota had a time limit for the isolation of diphtheria. In 8 it was two weeks, in 14, three, in 10, four, in 9, six, in 1, seven. In 12, two weeks and in 13, three weeks after recovery. I will refer to only one or two other objections to bacteriological quarantine. It is affirmed that where repeated cultures indicate the presence of Klebs-Löffler bacilli long after all trace of membrane has disappeared from the throat, these bacilli may not be virulent, or while morphologically identical with Klebs-Löffler bacilli, they may differ from them in producing few if any pathological lesions.

Unfortunately, animal inocculation in every case of suspected non-virulence is not practicable, but it is a significant fact that when the test has been applied in long standing cases the bacilli have been found virulent in nearly all. It would seem that the chance of unnecessary quarantine on this account is very small. It is also urged that virulent bacilli are sometimes found in the throats of persons in perfect health. This is true, but recent investigations by Kober would seem to show that this is the case much less frequently than is generally supposed.

Cultures were made from the throats of 128 individuals who had been in contact with cases of diphtheria, and 600 who had not been knowingly exposed to the disease. Bacilli were found in 8 per cent. of the first series and 15 or 2 5 per cent. of the second series.

Upon further inquiry it was found that 10 of the 15 in the second series could be considered to have been exposed directly or indirectly. This would leave 83-100 of 1 per cent. as the number of unexposed in whose throats Klebs-Löffler bacilli were found. The bacilli in all the cultures of the first series were found by animal inoculation to be virulent.

While it doubtless requires the presence of Klebs-Loffler bacilli, plus a certain susceptibility to the toxin which they produce to constitute a case of diphtheria, clinically speaking, it requires the presence of the virulent bacilli only to constitute a case capable of spreading the disease.

Bacilli may occasionally be found in the throats of healthy ndividuals, but it can not I think be argued from that fact that the continued quarantine of those who have recently had diphtheria, and in whose throats bacilli are found, is either unnecessary or unjust.

That there is the possibility of error in the bacteriological diagnosis and quarantine of diphtheria can not be denied, but is it too much to affirm that by its adoption a substantial advance has been made in the direction of early and accurate diagnosis and efficient isolation of this dread disease?

720 South Avenue.

